# Identification and characterization of four *Drosophila suzukii* cellularization genes and their promoters

**DOI:** 10.1186/s12863-020-00939-y

**Published:** 2020-12-18

**Authors:** Ying Yan, Syeda A. Jaffri, Jonas Schwirz, Carl Stein, Marc F. Schetelig

**Affiliations:** 1 Justus-Liebig-University Giessen, Institute for Insect Biotechnology, Department of Insect Biotechnology in Plant Protection, Winchesterstr. 2, 35394 Giessen, Germany; 2 Fraunhofer Institute for Molecular Biology and Applied Ecology IME, 35394 Giessen, Germany

**Keywords:** Spotted-wing Drosophila, Sterile insect technique, Transgenic embryonic sexing system, *D. melanogaster* S2 cells, Tetracycline-off system

## Abstract

**Background:**

The spotted-wing Drosophila (*Drosophila suzukii*) is a widespread invasive pest that causes severe economic damage to fruit crops. The early development of *D. suzukii* is similar to that of other Drosophilids, but the roles of individual genes must be confirmed experimentally. Cellularization genes coordinate the onset of cell division as soon as the invagination of membranes starts around the nuclei in the syncytial blastoderm. The promoters of these genes have been used in genetic pest-control systems to express transgenes that confer embryonic lethality. Such systems could be helpful in sterile insect technique applications to ensure that sterility (bi-sex embryonic lethality) or sexing (female-specific embryonic lethality) can be achieved during mass rearing. The activity of cellularization gene promoters during embryogenesis controls the timing and dose of the lethal gene product.

**Results:**

Here, we report the isolation of the *D. suzukii* cellularization genes *nullo, serendipity-α, bottleneck* and *slow-as-molasses* from a laboratory strain. Conserved motifs were identified by comparing the encoded proteins with orthologs from other Drosophilids. Expression profiling confirmed that all four are zygotic genes that are strongly expressed at the early blastoderm stage. The 5′ flanking regions from these cellularization genes were isolated, incorporated into *piggyBac* vectors and compared in vitro for the promoter activities. The *Dsnullo* promoter showed the highest activity in the cell culture assays using *D. melanogaster* S2 cells.

**Conclusions:**

The similarities in the gene coding and 5′ flanking sequence as well as in the expression pattern of the four cellularization genes between *D. melanogaster* and *D. suzukii*, suggest that conserved functions may be involved in both species. The high expression level at the early blastoderm stage of the four cellularization genes were confirmed, thus their promoters can be considered in embryonic lethality systems. While the *Dsnullo* promoter could be a suitable candidate, all reported promoters here are subject to further in vivo analyses before constructing potential pest control systems.

**Supplementary Information:**

The online version contains supplementary material available at 10.1186/s12863-020-00939-y.

## Background

The spotted-wing Drosophila (*Drosophila suzukii* Matsumura) is native to eastern Asia but has become a widespread invasive pest of fruit crops [1, 2]. The sterile insect technique (SIT) is a bio-control strategy that works by releasing a large number of typically radiation-sterilized males in the environment so they can compete with wildtype males. Mating between sterilized males and wildtype females produces no offspring, so the repeated release of sterile males leads to population suppression or eradication [3–5]. SIT has been used to control tephritid fruit flies, notably the Mediterranean fruit fly (*Ceratitis capitata*), with great success [4]. Consequently, it is also proposed as a cost-effective and environment-friendly control strategy for *D. suzukii* [6, 7]. Since sterilized females would compete with wild-type females for sterilized males and would still cause damage to fruit, *C. capitata* genetic sexing strains (GSS) have been developed to achieve male-only release [8], which is far more efficient and cost-effective than bisexual releases in the field [9, 10]. These GSSs use recessive pupal color or temperature sensitive lethal (tsl) mutations and Y:autosome translocations thus sexing can be achieved based on the pupal color or temperature-controlled female lethality [8]. However, it is difficult to transfer genetic sexing systems to other pest species because suitable recessive mutations and chromosome rearrangements are required in the target species.

Transgenic embryonic sexing systems (TESS) have been developed in several agricultural pests to enable male-only releases by killing all females at the embryonic stage [11–14]. The general mechanism involves the incorporation of binary tetracycline-off (Tet-off) modules into transposon vectors for germ-line transformation. Typically, the promoters of endogenous cellularization genes are used to express the *tetracycline transactivator* (*tTA*) gene (driver). In the absence of tetracycline, tTA binds to the tetracycline response element (TRE) and induces the TRE-linked pro-apoptotic gene (effector) only in females due to the presence of a sex-specific intron. Eliminating females before the feeding stage could reduce the insect diet cost during mass rearing thus lead to a more cost-effective SIT program.

A cellularization gene promoter is a key component of an efficient TESS because it controls *tTA* expression and determines the timing and dose of effector gene expression [13, 15]. There are four zygotic genes required for cellularization of the syncytial blastoderm in *D. melanogaster*: *nullo*, *serendipity-α* (*sry-α*), *bottleneck* (*bnk*) and *slow-as molasses* (*slam*) [16]. A 95-bp 5′ flanking sequence containing four conserved motifs is both necessary and sufficient for the blastoderm-specific expression of *Dm_sry-α* [17, 18]. The promoter activity at pre-blastoderm stage in *D. melanogaster* may also be controlled by TAGteam motifs, a series of 7-bp sequences commonly found in the 5′ flanking region of *D. melanogaster* genes expressed during early development [19]. The *nullo* and *sry-α* promoters have been used successfully to drive *tTA* and induce embryonic lethality in *D. melanogaster* [20]. In tephritid fruit flies, the *sry-α* promoter has been used most widely, and TESS systems incorporating this promoter have been reported in *Anastrepha suspensa* [11], *C. capitata* [12] and *Anastrepha ludens* [21]. The *bnk* and *nullo* promoters were used in the calliphorid blowfly *Lucilia cuprina* [13, 14]. Thus far, the *slam* promoter has not been used to develop functional TESS strains , possibly due to its low activity at the embryonic stage [12, 22]. However, a TESS promoter must not be too active, if the Tet-Off system is used, because high levels of *tTA* expression are also deleterious [23]. Therefore, systematic evaluation of cellularization genes and their promoters could facilitate the development of optimal TESS systems.

Here we identified four *D. suzukii* cellularization genes (*nullo, sry-α*, *bnk* and *slam*) based on the reference genome sequence in the Spotted Wing Drosophila database, SWDbase [24]. We isolated the coding sequence (CDS) of each gene from our laboratory strain (*D. suzukii* USA) and confirmed the presence of conserved motifs by comparing the encoded proteins with orthologs in other *Drosophila* species. The expression profile of each gene during development was verified by reverse transcription (RT)-PCR and quantitative real-time (qRT) PCR. We then isolated the 5′ flanking sequences from the four genes and incorporated them into *piggyBac* vectors for in vitro evaluation. Based on the promoter activity of these genes in insect cells, we discuss their potential applications in biocontrol strategies for *D. suzukii*.

## Results

### *D. suzukii* cellularization genes

**Fig. 1:**
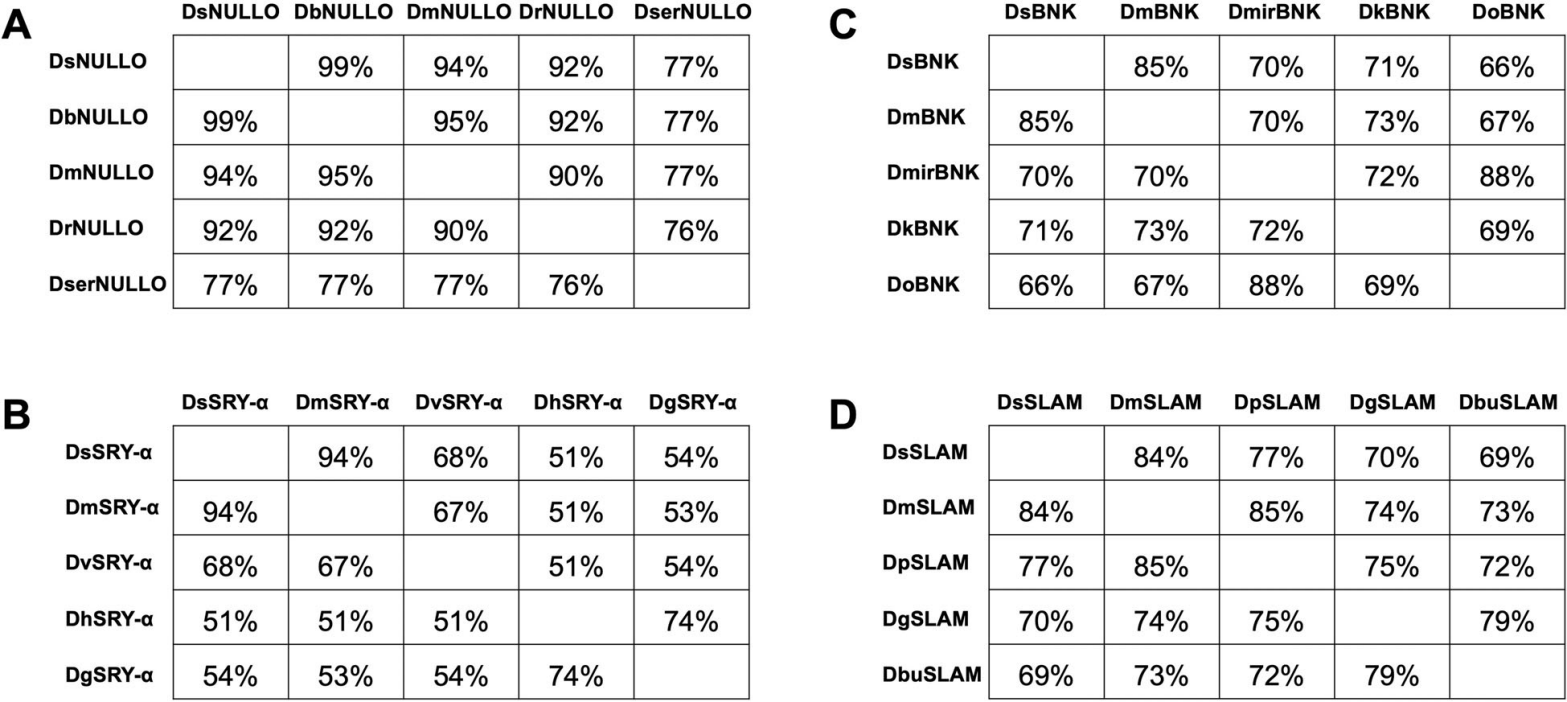
Similarities in the NULLO, SRY-α, BNK and SLAM proteins from *Drosophila spp*. **a** *Drosophila suzukii* NULLO (DsNULLO) is aligned with orthologs from *Drosophila biarmipes* [DbNULLO] (GenBank: XP_016948821.1), *Drosophila melanogaster* [DmNULLO] (GenBank: NP_511067.3), *Drosophila rhopaloa* [DrNULLO] (GenBank: XP_016982709.1), and *Drosophila serrata* [DserNULLO] (GenBank: XP_020805612.1). **b** *Drosophila suzukii* SERENDIPITY-α (DsSRY-α) is aligned with orthologs from *Drosophila grimshawi* [DgSRY-α] (GenBank: XP_001995347.1), *Drosophila hydei* [DhSRY-α] (GenBank: XP_023168708.1), *Drosophila melanogaster* [DmSRY-α] (GenBank: NP_524580.1) and *Drosophila virilis* [DvSRY-α] (GenBank: XP_002056142.1). **c** *Drosophila suzukii* BOTTLENECK (DsBNK) is aligned with orthologs from *Drosophila melanogaster* [DmBNK] (GenBank: NP_524604.2), *Drosophila miranda* [DmirBNK] (GenBank: XP_017140437.1), *Drosophila obscura* [DoBNK] (GenBank: XP_022213695.1), and *Drosophila kikkawai* [DkBNK] (GenBank: XP_017034088.1). **d** *Drosophila suzukii* SLOW-AS-MOLASSES (DsSLAM) is aligned with orthologs from *Drosophila grimshawi* [DgSLAM] (GenBank: XP_001988112.1), *Drosophila pseudoobscura* [DpSLAM] (GenBank: XP_001355861.2), *Drosophila melanogaster* [DmSLAM] (GenBank: NP_001285668.1), and *Drosophila busckii* [DbuSLAM] (GenBank: ALC38995.1). The protein alignment was performed in Geneious Prime program and similarities (Blosum45, threshold 1) are shown

Four *D. suzukii* cellularization genes were identified by homology searches in SWDbase and were compared in silico to known sequences. *Ds_nullo* (DS10_00005287) was identified as an intron-less gene like its *D. melanogaster* ortholog (*Dm_nullo*). The full-length 642-bp *Ds_nullo* CDS encodes a putative protein with 213 amino acids, most closely related to its ortholog in *D. biarmipes* (99% similarity, 96% identity), but also similar to its orthologs in *D. melanogaster* (94%) and *D. rhopaloa* (92%), with lower similarity (57–77%) in other Drosophilds (Fig. 1a; Additional file 2 Fig. S1). The N-terminus of DsNULLO features a myristoylation site and a cluster of positively charged amino acids, which may target the protein to the plasma membrane [25]. The remainder of the polypeptide features five conserved segments (Additional file 2 Fig. S1) required for NULLO proteins to stabilize the basal junction components in the nascent cleavage furrows of blastoderm cells [26].

*Ds_sry-α* (DS10_00012897) has three exons. The full-length 1593-bp *Ds_sry-α* CDS encodes a putative protein with 530 amino acids, most closely related to its ortholog in *D. melanogaster* (94% similarity, 87% identity; Fig. 1b). The N-terminus of DsSRY-α features a cysteine-rich motif, possibly a transmembrane segment (Additional file 2 Fig. S2). A conserved C-terminal region shows high similarity to Ezrin, Radixin and Moesin (ERM) proteins which facilitate actin–membrane interactions [27], suggesting DsSRY-α fulfils a similar role in the reorganization of microfilaments during cellularization [28].

**Fig. 2:**
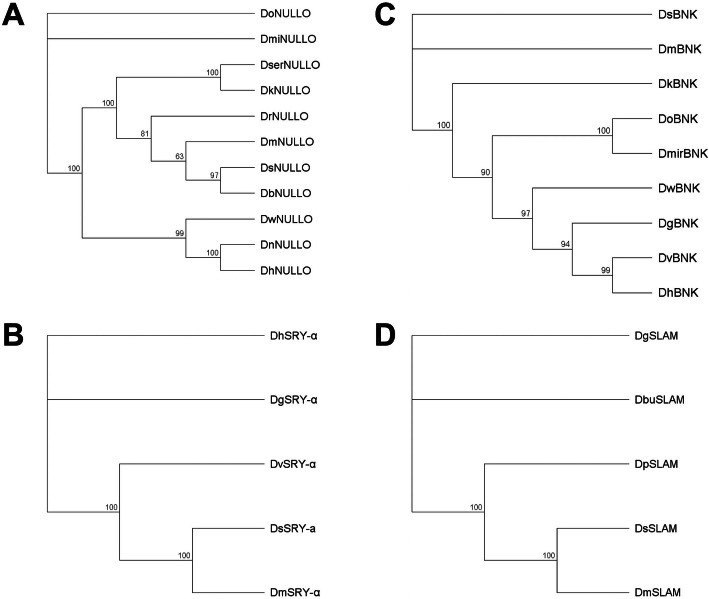
Phylogenetic analysis of *Drosophila* spp. NULLO, SRY-α, BNK and SLAM proteins. Unrooted neighbor-joining trees were constructed with amino acid sequences from **a** NULLO, **b** SRY-α, **c** BNK, and **d** SLAM. Bootstrap values (1000 replicates) are shown on the nodes of the trees. In addition to the orthologs in Fig. 1a, *Drosophila hydei* [DhNULLO] (GenBank: XP_023161247.1), *Drosophila kikkawai* [DkNULLO] (GenBank: XP_017022675.1), *Drosophila miranda* [DmirNULLO] (GenBank: XP_017156218.1), *Drosophila navojoa* [DnNULLO] (GenBank: XP_017964581.1), *Drosophila obscura* [DoNULLO] (GenBank: XP_022210380.1), and *Drosophila willistoni* [DwNULLO] (GenBank: XP_002071148.1) are used for the alignment. In addition to the orthologs in Fig. 1c, *Drosophila grimshawi* [DgBNK] (GenBank: EDV90810.1), *Drosophila hydei* [DhBNK] (GenBank: XP_023168586), *Drosophila virilis* [DvirBNK] (GenBank: XP_002055918.1) and *Drosophila willistoni* [DwBNK] (GenBank: XP_002072000.1) are used for the alignment. Other species abbreviations are the same as in Fig. 1

*Ds_bnk* (DS10_00007356), like *Ds_nullo*, is an intron-less gene. The 903-bp *Ds_bnk* CDS encodes a putative protein with 303 amino acids, most closely related to its ortholog in *D. melanogaster* (85% similarity, 80% identity; Fig. 1c). Finally, *Ds_slam* (DS10_00010822) has two exons. The 3414-bp *Ds_slam* CDS encodes the largest of the four cellularization proteins, with 1137 amino acids. DsSLAM is most closely related to its ortholog in *D. melanogaster* (88% similarity, 74.6% identity; Fig. 1d). Previous studies have not identified any functional motifs in DmBNK [29] or DmSLAM [30]. However, several highly conserved regions were identified in DsBNK (Additional file 2 Fig. S3) and DsSLAM (Additional file 2 Fig. S4). The phylogenetic analysis of all four proteins using a neighbor-joining algorithm revealed that DsSRY-α, DsBNK and DsSLAM clustered with their *D. melanogaster* orthologs, whereas DsNULLO clustered with DbNULLO and DmNULLO (Fig. 2).

### *D. suzukii* cellularization genes are strongly expressed during embryogenesis

**Fig. 3:**
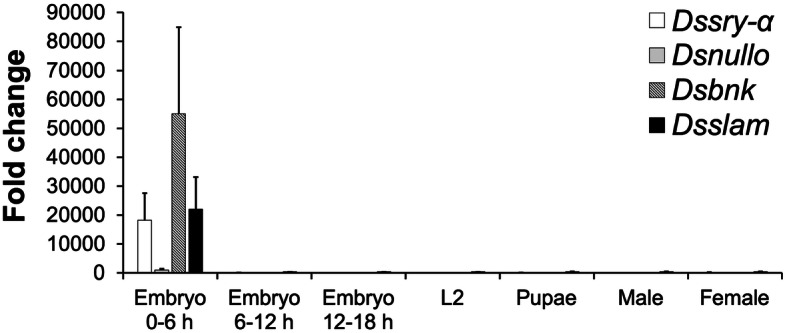
Quantitative real-time-PCR to determine relative expression of cellularization genes. Three different embryonic stages (0–6 h, 6–12 h and 12–18 h after egg laying), the second instar larvae (L2), pupae and 5 d old males and females were analyzed. The gene expression was normalized to the reference genes *TBP* and *AK*, and related to the *Dsnullo* expression level at 6–12 h embryos. The mean and standard error from three replicate experiments are shown

The RT-PCR results suggest that mRNA levels of all four cellularization genes were undetectable during the 0–0.5 h time window, reached a sharp peak around the onset of cellularization (2–3 h after egg laying) and then declined, but nevertheless persisted to the adult stage (Additional file 2 Fig. S5). *Ds_sry α* expression peaked slightly later than *Ds_nullo*, as previously reported for the *D. melanogaster* orthologs [31]. The *Ds_bnk* and *Ds_slam* transcripts were particularly abundant at the 1–3 h stage, but declined sharply thereafter (Additional file 2 Fig. S5). Primers for the reference genes *TATA binding protein* (*TBP*)*, glyceraldehyde-3-phosphate dehydrogenase* (*GAPDH*)*, arginine kinase* (*AK*)*, a-Tubulin* (*α-tub*) and *Histone H3* (*His3*) were also evaluated for their performance using RT-PCR analysis [32, 33]. The primers that amplified products from most or all stages (Additional file 2 Fig. S5) were further evaluated for efficiency using a serial dilution of cDNA as templates. The primer efficiency was calculated as 99.4% for *Ds_TBP*, 93.6% for *Ds_GAPDH*, 99.5% for *Ds_AK*, and 89.6% for *Ds_His3*. Consequently, primers for *Ds_TBP* and *Ds_AK* that showed high efficiency were used in the qRT-PCR experiments. The qRT-PCR data were consistent with our semi-quantitative RT-PCR analysis, with all four cellularization genes expressed at the highest level during the early blastoderm stages (Fig. 3). When normalized with *Ds_TBP* and *Ds_AK*, the *Ds_sry-α*, *Ds_nullo*, *Ds_bnk* and *Ds_slam* transcripts were 123-, 1040-, 13,469- and 56-fold more abundant in 0–6 h embryos than 6–12 h embryos, and 116-, 153-, 2433- and 49-fold more abundant in 0–6 h embryos than adult female, respectively (Fig. 3). Furthermore, in 0–6 h embryos the *Dssry-α*, *Dsbnk* and *Dsslam* transcripts were respectively 17.5-fold, 52.9-fold and 21.2-fold more abundant than *Dsnullo* mRNA. The relatively large variations (error bars) in gene expression from 0 to 6 h embryos are possibly due to the different ratios of eggs collected at early blastoderm stages (2–3 h) to eggs from other time period within 0–6 h from three replicates. Nevertheless, the expression levels of all four genes were much lower at later developmental stages (Fig. 3).

### 5′ flanking region of *D. suzukii* cellularization genes

**Fig. 4:**
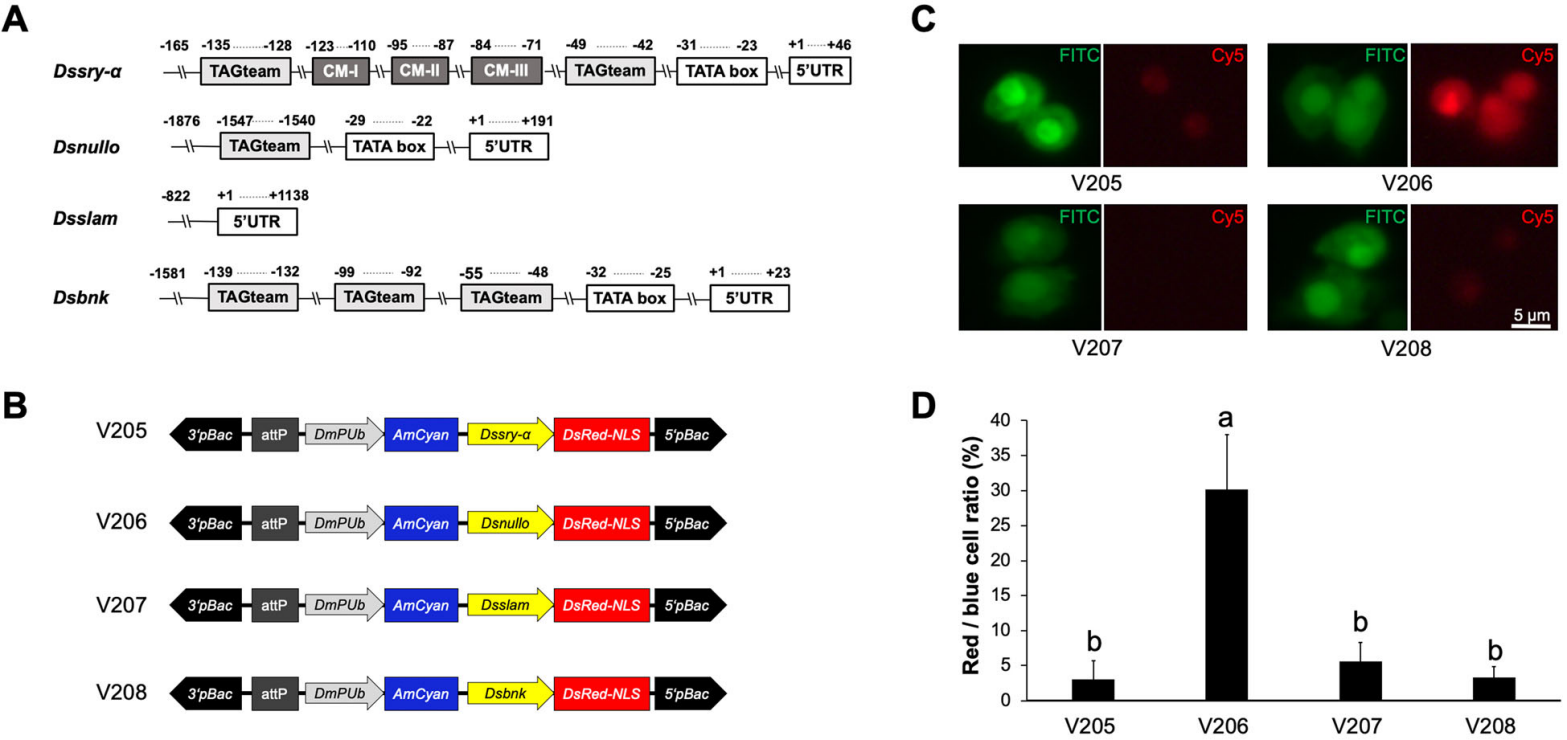
Functional characterization of *Drosophila suzukii* cellularization gene promoters. **(a)** Schematic map of the 5′ flanking sequences from four cellularization genes (*Dssry-α, Dsnullo, Dsslam* and *Dsbnk*). The positions of TAGteam motifs, conserved motifs (CM), TATA box and 5′ UTR were indicated. **(b)** *piggyBac* test vectors V205, V206, V207 and V208, in which the *Dssry-α*, *Dsnullo*, *Dsslam* and *Dsbnk* promoters, are used to drive the expression of DsRed with a nuclear localization signal (NLS), respectively. All test vectors contain an AmCyan marker gene regulated by the constitutive *D. melanogaster polyubiquitin* (*DmPUb*) promoter and an *att*P recombination site. **(c)** *Drosophila* S2 cells were transfected with *piggyBac* vectors and images taken under epifluorescent light conditions using the FITC filter for AmCyan or CY5 filter for DsRed. **(d)** The number of cells that show red and blue fluorescence was counted using Image J, and the ratio of red to blue cells was calculated. Each bar presents the mean ± SE of *n* = 3 experiments. The bars with different letters are significantly different at *P* < 0.05 (one-way ANOVA, Duncan’s multiple range test)

The 5′ flanking region of *Ds_sry-α* lacked motif IV but contained two TAGteam motifs, as well as a TATA box (TATATAAA) 23 bp upstream of the putative transcription start site (TSS) (Fig. 4A; Additional file 2 Fig. S6). The 5′ flanking region of *Ds_nullo* contained one TAGteam motif and a TATA box (TATATAT) 24 bp upstream of the predicted TSS (Fig. 4A; Additional file 2 Fig. S7). The 5′ flanking region of *Ds_bnk* contained three TAGteam motifs and a TATA box (TATATAAA) 25 bp upstream of the TSS (Fig. 4A; Additional file 2 Fig. S8). However, neither of 5′ flanking sequences of *Ds_slam* and *Dm_slam* contained TAGteam motifs; there was also no TATA box in these regions identified (Fig. 4A; Additional file 2 Fig. S9). We prepared four *piggyBac* test vectors (V205–V208) in which the 5′ flanking sequence of *Ds_sry-α*, *Ds_nullo*, *Ds_slam* or *Ds_bnk* was linked to the *DsRed* reporter gene, respectively (Fig. 4B). All vectors also contained a *DmPUb*-*AmCyan* marker, allowing us to use an EGFP filter to determine the efficiency of transfection. We transfected *D. melanogaster* S2 cells with each of the four constructs, fixed the cells 18 h post-transfection and counted the numbers of blue and red fluorescent cells. The ratio of red to blue cells therefore provided an internally-consistent readout of regulation activity from the 5′ flanking sequence. We found that the 5′ flanking sequence of *Ds_nullo* (construct V206) generated the strongest DsRed signal (Fig. 4C) and the highest DsRed:AmCyan ratio (Fig. 4D) indicating the highest activity as a gene promoter (*P* = 0.001 when compared to V205 and V208, *P* = 0.002 when compared to V207, one-way ANOVA).

## Discussion

Early development in many insect species involves a syncytial blastoderm stage followed by cellularization after a certain number of nuclear divisions, and this process is orchestrated by a small number of zygotic genes [16]. Four major cellularization genes have been identified in the developmental model organism *D. melanogaster* (*nullo*, *sry-α*, *bnk* and *slam*), and the conserved sequences of the orthologs in *D. suzukii* (Additional file 2 Fig. S1–4) together with the peak of expression at the early blastoderm stage (Fig. 3) suggest that similar functions may be involved in both species, including the regulation of actin filaments, microfilaments and membrane polarization [16]. *D. melanogaster* embryogenesis has been divided into 17 stages, with the cellular blastoderm arising during stage 5, 2 h 10 min and 2 h 50 min after egg laying [34]. During this stage, *nullo*, *sry-α*, *bnk,* and *slam* are expressed to coordinate the cellularization process, but expression declines thereafter, to low levels for *nullo* and *bnk*, and to moderate levels for *sry-α* and *slam* [35]. In many fly species, the expression of all four cellularization genes is typically detected as early as 1–2 h after egg laying [13, 22, 31]. However, the corresponding transcripts were already present in *D. suzukii* embryos collected 0–1 h after egg laying, suggesting that gene expression commences at the start of embryogenesis (Additional file 2 Fig. S5). This apparent early expression may reflect the facultative ovoviviparity of *D. suzukii* females, allowing them to retain fertilized eggs, which can therefore develop to a certain extent before laying [36]. However, the transcripts of all four cellularization genes were undetectable during the 0–0.5 h time window (Additional file 2 Fig. S5), confirming the absence of maternal gene expression and that egg retention was not responsible for the early transcripts – instead, the early zygotic gene expression was a genuine phenomenon.

The early and stage-specific expression is important for the development of genetic pest-control systems because it allows us to focus the effect of lethal effector transgenes at the correct developmental stage [11, 13, 37]. Specifically, constitutive and unconditional effector gene expression later in development could lead to undesired female sterility or lethality in a control strain and has been reported previously in interfering with ovarian development and reducing productivity of those strains [14, 15, 21]. Our qRT-PCR data showed that the four *D. suzukii* cellularization genes were expressed with a similar profile to their orthologs in *D. melanogaster* (Fig. 3). The considerably higher gene expression in 0–6 h embryos than 6–12 h embryos for *Ds_nullo* and *Ds_bnk* (1040-fold and 13,469-fold, respectively), suggesting that their promoters are likely early active and would therefore be useful candidates for the development of TESS systems in *D. suzukii*, whereas the *Ds_sry-α* and *Ds_slam* promoter may allow moderate but constitutive expression at later developmental stages. Therefore, those early-embryonic promoters could be used to establish *D. suzukii* sexing systems for SIT programs in the future. Multiple promoters and independent systems will also be important in dealing with natural resistance processes [38]. Those backup systems would help establish stable lines that overcome primary-site mutations during rearing processes and quality control of the mass-reared strains, and help strains to cope with second-site maternal-effect suppressors [38].

The 5′ flanking region of the *D. suzukii* cellularization genes contained several TAGteam motifs, which are often found in *D. melanogaster* genes expressed early in development, and act as binding sites for the transcriptional activator Zelda [19, 39]. The 5′ flanking region of *Ds_bnk* contains three TAGteam motifs 91–139 bp upstream of the predicted TSS, whereas that of *Ds_sry-α* contains two such motifs 93–135 bp upstream of the predicted TSS (Fig. 4A). In contrast, the 5′ flanking region of *Ds_nullo* features only one TAGteam motif, which is much further away from the TSS: 1576 bp upstream (Fig. 4A). Such differences in the number and location of TAGteam motifs may contribute to differences in gene expression at the 0–6 h embryonic stage, since *Ds_bnk* and *Ds_sry-α* mRNA were more than 10-fold more abundant than *Ds_nullo* mRNA (Fig. 3). Interestingly, neither the *Ds_slam* nor the *Dm_slam* 5′ flanking region feature any TAGteam motifs (Fig. 4A; Additional file 2 Fig. S9) but *Ds_slam* was nevertheless expressed at relatively high levels during the 0–6 h embryonic stage. This suggests that the transcriptional activation of *Ds_slam* may be less dependent on Zelda compared to the other genes.

*piggyBac*-based vectors have been used to evaluate the embryonic activity of the promoters using the *A. suspensa* cell line UFENY-AsE01, an embryonic cell line derived originally from 20-h old embryos [40]. Previously, *sry-a* promoters from *C. capitata* and *A. suspensa* were able to mediate cell death in these AsE01 cells as well as embryonic lethality in transgenic flies [11, 37, 41]. In addition, the *D. melanogaster* S2 cells are also derived from late embryonic stages [42], and have been one of the few *D. melanogaster* cell lines that can be used for heterologous gene expression [43]. Often high levels of heterologous protein expression in S2 cells are due to strong promoters in the expression vectors [44, 45]. Although *Ds_nullo* was expressed at the lowest level among the four genes at the 0–6 h embryonic stage, its promoter achieved the strongest DsRed expression in the cell culture tests (Fig. 4C, D). Such observation suggested that the *Ds_nullo* promoter mediated higher protein production than other tested promoters in the embryonic S2 cells. The weaker activity of *Ds_sry-α* promoter may be due to the absence of motif IV, which was suggested to function as a positive *cis*-acting regulatory element. Previous reports verified that the deletion of motif IV in the *Dm_sry-α* promoter resulted in a more than four-fold loss of activity at the blastoderm stage [18]. Weak transgene expression was also observed for the *slam* promoters from *C. capitata* [12] and *L. cuprina* [22], which do not have TATA box motifs. Similarly, both *Dm_slam* and *Ds_slam* promoter sequences lack TATA box motifs (Additional file 2 Fig. S9). Such TATA-less genes often show multiple TSSs [46], and are more flexibly regulated compared to TATA-containing genes [47]. Thus, *slam* in these species may involve multiple elements for its correct regulation. Computational and functional studies in other species revealed an importance for transcriptional activity based on motifs in the core promoter sequence [48] and a number of relevant transcription factors involved have been studied [47]. But ultimately, the reported promoters have to be functionally tested to evaluate their potential for pest control applications.

## Conclusions

For *D. melanogaster*, the gene networks that regulate early development were intensively studied to understand the basic biological processes such as cellularization, sex determination and patterning [16, 19, 39, 48]. Here we have verified the similarities and differences in the promoter and CDS sequences of four cellularization genes between *D. melanogaster* and *D. suzukii.* More functional studies are needed at transcription or protein level [17, 29–31] to understand the gene interaction and network connection during early development in *D. suzukii*. In addition, *D. melanogaster* S2 cells are potentially a good system to study early genes and their promoters due to its embryonic origin [42]. Our results indicated that the *Ds_nullo* promoter can be considered for driving embryonic lethality in a genetic control system such as a TESS. Nevertheless, all reported promoters here have to be further functionally analysed to evaluate their in vivo performance before constructing potential pest control systems.

## Methods

### Insect rearing and sample collection

Wild-type *D. suzukii* USA specimens were maintained at 25 °C and 60% humidity with a 12-h photoperiod. Eggs were collected over a 30-min or 1-h period on grape juice agar plates as previously described [49]. We allowed the eggs to develop to the desired developmental time point before freezing them in liquid nitrogen. The larvae and pupae were directly collected from stock vials at the desired stage. Adult males and females were isolated immediately after eclosion and sampled 1 or 5 days later.

### Gene sequence isolation and analysis

The *gene query* function was not available in SWDbase [24] when this work was initiated in 2014. Therefore, the CDS of the *D. melanogaster* genes *Dm_sry-α* (FBgn0003510), *Dm_nullo* (FBgn0004143), *Dm_bnk* (FBgn0004389) and *Dm_slam* (FBgn0043854) were obtained from FlyBase (http://flybase.org/) and were used as tBLASTx queries against SWDbase. The *Ds_sry-α* query matched a 1593-bp CDS on scaffold 705. *Ds_nullo* matched a 642-bp CDS on scaffold 5. *Ds_bnk* matched a 903-bp CDS on scaffold 21. Finally, *Ds_slam* matched a 1677-bp CDS on scaffold 181. Based on these hits, primers were designed to amplify the full-length CDS of each gene from cDNA prepared from wild-type *D. suzukii* USA embryos (Additional File 1). Total RNA was isolated from embryos at the early blastoderm stage (3–4 h after egg laying) using the ZR Tissue & Insect RNA MicroPrep kit (Zymo Research) and residual DNA was removed with Turbo DNAse (Ambion). The iScript cDNA Synthesis Kit (Bio-Rad) was used to prepare cDNA from 1 μg DNA-free RNA. For *Ds_bnk*, *Ds_slam,* and *Ds_nullo*, each 25-μl reaction mix comprised 0.2 μL Platinum Taq DNA polymerase (Invitrogen), 2.5 μL 10x PCR buffer, 0.75 μL 50 mM MgCl_2_, 1.0 μL 10 mM dNTP mix, 1.0 μL of each primer (0.4 μM), and 0.5 μL cDNA. An initial denaturation step at 95 °C for 2 min was followed by 35 cycles of denaturation at 95 °C for 30 s, annealing at 52 °C for 30 s and extension at 72 °C for 1 min (*nullo* and *bnk*) or 2 min (*slam*), and a final extension at 72 °C for 5 min. For *Ds_sry-α*, multiple PCR attempts using the Platinum Taq DNA polymerase did not result in amplificates, thus the Phusion Flash High-Fidelity DNA Polymerase (Thermo Fisher Scientific) was used. PCR was carried out using 10 μL of the Phusion Flash High-Fidelity PCR Master mix with 1.0 μL of each primer and 1.0 μL cDNA (0.4 μM) in a 25-μl reaction. An initial denaturation step at 98 °C for 10 s was followed by 30 cycles of denaturation at 98 °C for 30 s, annealing at 52 °C for 5 s and extension at 72 °C for 30 s, a final extension at 72 °C for 1 min, and a 4 °C hold. The PCR products were separated by 1% agarose gel electrophoresis and extracted from the gels using the QIAquick Gel Extraction Kit (Qiagen) before cloning in vector pCR4-TOPO. The presence of inserts was confirmed by restriction digestion with EcoRI and sequencing using M13 primers. Sequence translation, polypeptide alignment and phylogenetic analysis were performed using the Geneious Prime software [50].

### Gene expression analysis by RT-PCR and qRT-PCR

The RT-PCR was assembled using platinum Taq polymerase as described above. The reaction profile comprised an initial denaturation step at 94 °C for 4 min, followed by 35 cycles of denaturation at 94 °C for 30 s, annealing at 55 °C (*His3*, *TBP*, *nullo*, *bnk* and *slam*) or 60 °C (*AK, GADPH, α-Tub*, *sry-α*) for 30 s, and extension at 72 °C for 1 min, and a final extension at 72 °C for 4 min. We used SsoAdvanced Universal SYBR Green Supermix (Bio-Rad) with 100 ng of template cDNA for the qRT-PCR experiments. The reactions were performed in a CFX96 Touch Real-Time PCR Detection System (Bio-Rad) and each comprised an initial denaturation step at 95 °C for 30 s, followed by 40 cycles of denaturation at 95 °C for 10 s, annealing at 60 °C for 20 s, extension at 65 °C for 5 s, and a machine related 95 °C for 0.5 s step. All experiments were carried out in three biological triplicates each with three technical replicates. The 2^-∆∆Ct^ formula was used for all samples and values were normalized to the geometric mean of the reference genes *TBP* and *AK*. All primer sequences were listed in Additional file 1.

### Promoter isolation and plasmid construction

The upstream flanking sequences of the *D. melanogaster* and *D. suzukii* cellularization genes were obtained from FlyBase and SWDbase, respectively. The sequences were aligned using Geneious and searched for conserved motifs, TAGteam motifs [19] and the TATA box. High-molecular-weight genomic DNA was prepared from *D. suzukii* adults using DNAzol Reagent (Thermo Fisher Scientific). Promoter fragments were amplified from genomic DNA using the primers listed in Additional file 1 and were transferred to pCR4-TOPO as described above. After confirming the nucleotide sequences, the promoter fragments were amplified and used to replace the *D. melanogaster polyubiquitin* (*DmPUb*) promoter in the *piggyBac* vector pXLBacII-attP-PUbAmCyan_DmPUb_DsRed-NLS-SV40 [51] at the Bsu36I and MluI restriction sites, to obtain the test vectors V205–V208.

### Cell culture experiments

*Drosophila* Schneider 2 (S2) cells [42] were grown on Schneider’s medium containing 10% heat-inactivated fetal bovine serum (Hi-FBS) and 1% penicillin/streptomycin in closed-capped flasks without CO_2_ at 25 °C. Cells were passaged every 2 days until ≥90% viability was achieved. For transient transfection, we used Xfectin transfection reagent (Takara) according to the manufacturer’s instructions. We placed a 13-mm TC coverslip (Sarstedt) into each well of a 24-well plate to facilitate imaging, then seeded each well with 5 × 10^5^ cells in 500 μL of medium. After 3 h, the settled cells were transfected using 2 μg of plasmid DNA, 0.6 μL Xfectin, 27.4 μL Xfectin buffer and 270 μL serum-free Schneider’s medium for 4 h. Transfection was stopped by refreshing the dishes with 500 μL Schneider’s medium containing 10% Hi-FBS and 1% penicillin/streptomycin. The cells were incubated for ~ 18 h at 25 °C before fixing in 4% paraformaldehyde for 15 min and washing briefly with PBS prior to microscopy.

### Cell imaging and counting

*Drosophila* S2 cells transfected with constructs V205–V208 were imaged using an inverted microscope equipped with a fluorescent slide containing the FITC (ex: 490/20; detecting also AmCyan expression) and CY5 (ex: 620/60) filters (Leica DM IL LED, Leica Microsystems, Wetzlar, Germany). All settings (e.g., exposure time, gain, magnification) were identical for each test so that the fluorescence intensity could be compared. Fluorescent cells were counted in Image J (Fiji) using the automated cell count function. Specifically, raw images were converted to an 8-bit standardized format and inverted before a watershed was applied to separate any cells in direct contact. The counting threshold was set to 30 for the DsRed filter. The differences in red:blue cell ratios (data were square root transformed) for different *piggyBac* vectors were tested by one-way analysis of variance (ANOVA) and means were separated using Duncan’s multiple range test in SigmaPlot v12.5 (Systat Software).

## Supplementary Information

**Additional file 1.** Primer sequences.

**Additional file 2:** **Figure S1.** *Drosophila suzukii* NULLO protein alignment. DsNULLO is aligned with orthologs from *Drosophila biarmipes* [DbNULLO] (GenBank: XP_016948821.1), *Drosophila hydei* [DhNULLO] (GenBank: XP_023161247.1), *Drosophila kikkawai* [DkNULLO] (GenBank: XP_017022675.1), *Drosophila melanogaster* [DmNULLO] (GenBank: NP_511067.3), *Drosophila miranda* [DmirNULLO] (GenBank: XP_017156218.1), *Drosophila navojoa* [DnNULLO] (GenBank: XP_017964581.1), *Drosophila ob*scura [DoNULLO] (GenBank: XP_022210380.1), *Drosophila rhopaloa* [DrNULLO] (GenBank: XP_016982709.1), *Drosophila serrata* [DserNULLO] (GenBank: XP_020805612.1) and *Drosophila willistoni* [DwNULLO] (GenBank: XP_002071148.1). Identical amino acids are shaded black and conservative changes are indicated in gray. All proteins contain a consensus site for N-terminal myristoylation (M) followed by a positively charged cluster (P). The remainder of the protein contains five conserved regions of amino acids (A–E) separated by short non-conserved regions. **Figure S2.** *Drosophila suzukii* SERENDIPITY-α (SRY-α) protein alignment. DsSRY-α is aligned with orthologs from *Drosophila grimshawi* [DgSRY-α] (GenBank: XP_001995347.1), *Drosophila hydei* [DhSRY-α] (GenBank: XP_023168708.1), *Drosophila melanogaster* [DmSRY-α] (GenBank: NP_524580.1) and *Drosophila virilis* [DvSRY-α] (GenBank: XP_002056142.1). The putative transmembrane domain is underlined and the region of similarity with proteins of the ERM family is boxed. **Figure S3.** *Drosophila suzukii* BOTTLENECK (BNK) protein alignment. DsBNK is aligned with orthologs from *Drosophila grimshawi* [DgBNK] (GenBank: EDV90810.1), *Drosophila hydei* [DhBNK] (GenBank: XP_023168586), *Drosophila kikkawai* [DkBNK] (GenBank: XP_017034088.1), *Drosophila melanogaster* [DmBNK] (GenBank: NP_524604.2), *Drosophila miranda* [DmirBNK] (GenBank: XP_017140437.1), *Drosophila obscura* [DoBNK] (GenBank: XP_022213695.1), *Drosophila virilis* [DvirBNK] (GenBank: XP_002055918.1) and *Drosophila willistoni* [DwBNK] (GenBank: XP_002072000.1). Highly conserved regions are marked with red lines. **Figure S4.** *Drosophila suzukii* SLOW-AS-MOLASSES (SLAM) protein alignment. DsSLAM is aligned with orthologs from *Drosophila grimshawi* [DgSLAM] (GenBank: XP_001988112.1), *Drosophila pseudoobscura* [DpSLAM] (GenBank: XP_001355861.2), *Drosophila melanogaster* [DmSLAM] (GenBank: NP_001285668.1), and *Drosophila busckii* [DbuSLAM] (GenBank: ALC38995.1). Highly conserved regions are marked with red lines. **Figure S5.** Reverse-transcriptase (RT)-PCR to evaluate the reference **(a)** and cellularization **(b)** genes through the development of *Drosophila suzukii*. Primer sequences for reference genes *TATA binding protein* (*TBP*), *glyceraldehyde-3-phosphate dehydrogenase* (*GAPDH*), *arginine kinase* (*AK*), *a-Tubulin* (*α-Tub*) and *Histone H3* (*His3*) (Zhai et al., 2014; Li and Handler, 2017), as well as for four cellularization genes can be found in “Additional file 1”. Embryos collected at different time points after egg laying (in hours), larvae (first, second and third instar), pupae (2 days after prepupae), adult female and male (1 and 5 days old). Additional 0–0.5 h samples were used for cellularization genes. The PCR product sizes are 140 bp for *AK*, 130 bp for *GAPDH*, 189 bp for *α-Tub*, 129 bp for *His3*, and 182 bp for *TBP*, 161 bp for *sry-a*, 155 bp for *nullo*, 159 bp for *bnk*, and 149 bp for *slam*. M is the molecular weight ladder. **Figure S6**. Alignment of the *Ds_sry-α* and *Dm_sry-α* 5′ flanking sequences. The upstream flanking sequence from *Ds_sry-α* (165 bp, before it reaches the upstream gene DS10_00012896, the ortholog of *D. melanogaster janus A*) and *Dm_sry-α* (167 bp) together with the 5′-UTR (annotated in green) are compared. The 5′ flanking sequences of *Ds_sry-α* and *Dm_sry-α* contain three and four conserved motifs that confer blastoderm-specific expression, respectively. Both 5′ flanking sequences contain two TAGteam motifs (annotated in yellow), and a TATA box (TATATAAA) 23 bp upstream of the putative transcription start site. The *Ds_sry-α* 5′ flanking sequence was fused to DsRed-NLS in V205 to act as a gene promoter for the in vitro test. **Figure S7**. Alignment of the *Ds_nullo* and *Dm_nullo* 5′ flanking sequences. The upstream flanking sequence from *Ds_nullo* (1876 bp) and *Dm_nullo* (1753 bp) together with the 5′-UTR (annotated in green) are compared. The 5′ flanking sequences of *Ds_nullo* sequence contains one TAGteam motif (annotated in yellow), whereas that of *Dm_nullo* contains none. The TATA box (TATATAT) is 24 bp upstream of the putative transcription start site. The *Ds_nullo* 5′ flanking sequence was fused to DsRed-NLS in V206 to act as a gene promoter for the in vitro test. **Figure S8**. Alignment of the *Ds_bnk* and *Dm_bnk* 5′ flanking sequences. The upstream flanking sequence from *Ds_bnk* (1581 bp) and *Dm_bnk* (1523 bp) together with the 5′ UTR (annotated in green) are compared. The 5′ flanking sequences of *Ds_bnk* and *Dm_bnk* contain three and four TAGteam motifs (annotated in yellow), respectively, and the TATA box (TATATAAA) is 25 bp upstream of the putative transcription start site. The *Ds_bnk* 5′ flanking sequence was fused to DsRed-NLS in V208 to act as a gene promoter for the in vitro test. **Figure S9**. Alignment of the *Ds_slam* and *Dm_slam* 5′ flanking sequences. The upstream flanking sequence from *Ds_slam* (822 bp) and *Dm_slam* (887 bp) together with the 5′ UTRs (annotated in green) are compared. Neither the *Ds_slam* nor *Dm_slam* 5′ flanking sequences contain TAGteam motifs or a TATA box. The *Ds_slam* 5′ flanking sequence was fused to DsRed-NLS in V207 to act as a gene promoter for the in vitro test.

## Data Availability

All data generated or analyzed during this study are included in this published article [and its supplementary information files]. The GenBank accession numbers are as follows: *Ds_sry-α* mRNA: MK392555; *Ds_nullo* mRNA: MK392556; *Ds_bnk* mRNA: MK392557; *Ds_slam* mRNA: MK392558.
