# Supplementary material for: Identification and characterization of four Drosophila suzukii cellularization genes and their promoters
Source: BMC Genet. 2020 Dec 18;21(Suppl 2):146. doi: 10.1186/s12863-020-00939-y (PMC7747377; doi:10.1186/s12863-020-00939-y)
Supplement: Supplementary file 1 — Additional file 1. Primer sequences. [file 12863_2020_939_MOESM1_ESM.pdf]

Additional file 1. Primer sequences

Primer	Primer name	Primer sequence (5′→3′)
P1	Dssry-a_P_F	CACCACCTGTTCCTGTAGC
P2	Dssry-a_P_R	GAAGTTATCGAGCTCAGGC
P3	Dsnullo_P_F	AGTTCTTTGCCTGTATTTCAGGAACTC
P4	Dsnullo_P_R	TGCGATCAGGTCACTGCAAG
P5	Dsbnk_P_F	GCTCACAGGGAATGGATTCAGAG
P6	Dsbnk_P_R	TGGTCGAGTCCTCTCCGCTC
P7	Dsslam_P_F	AAGGAGGTGGAGCAATGAGAC
P8	Dsslam_P_R	TCAGCTTGTAGTTGTAGAACTGGAAG
P462	Bsu_Dssry-a_P_F	ACGTACCCTGAGGAGTTCTTTGCCTGTATTTCAGG
P463	Mlu_Dssry-a_P_R	ACGTACACGCGTAGCTCTATAAGATGTGCTCCCGG
P464	Bsu_Dsnullo_P_F	ACGTACCCTGAGGGATGCTCTTTTAGGAATTTATC
P465	Mlu_Dsnullo_P_R	ACGTACACGCGTTTTTCTCAAAACTTAGGTT
P466	Bsu_Dsbnk_P_F	ACGTACCCTGAGGTAGAATCTGCATGCCTAATC
P467	Mlu_Dsbnk_P_R	ACGTACACGCGTTTTAAGACCACTCTTTTTCTG
P468	Bsu_Dsslam_P_F	ACGTACCCTGAGGAGGAGGTGGAGCAATGAG
P469	Mlu_Dsslam_P_R	ACGTACACGCGTGTTGAAGGCTGATAAACGA
P1370	Dzbnk-ORF-F	ATGAGCATCAGCACTTTCAACTTCCAG
P1371	Dzbnk-ORF-R	TTAGGCACTCATTGAGATGCGTTGC
P1372	Dznullo-ORF-F	ATGGGCAGCACTCATTCCGCTG
P1373	Dznullo-ORF-R	CTAGATCTTCACCAGTCGTTCGCGG
P1374	Dzslam-ORF-F1	ATGGTTGTAAACACCGCAGCCATG
P1375	Dzslam-ORF-R1	CTATACCTCCACGGCCCTTCGG
P1348	Dzslam-ORF-F2	GCTGCAAGCCATCCGAAGC
P1223	Dzslam-ORF-R2	GCGTCTCGGGCTGAGATTCGTCGC
P712	Dz-GADPH-qF	GATCACCGTCTTCAGCGAAC
P713	Dz-GADPH-qR	GGCACCGCCCTTCAAGTG
P738	Dz-α-tub-qF	AGGATGCGGCGAATAACT
P739	Dz-α-tub-qR	CGGTGGATAGTCGCTCAA
P740	Dz-TBP-qF	CCACGGTGAATCTGTGCT
P741	Dz-TBP-qR	GGAGTCGTCCTCGCTCTT
P1234	Dz-AK-qF	ACGGTGAACCCAATGGCACCGC
P1235	Dz-AK-qR	CAACAGCGACTTGGAGTCGGAGGC
P1360	Ds_His3_qF	GAACGGTTGCCCTGCGTG
P1361	Ds_His3_qF	AGCTCTGGAATCGCAGGTCAG
P1562	Dz_slam_qF	CACGCTGCAGATATCAAGGC
P1563	Dz_slam_qR	GGATACACTTTGCCTCCAGTTCC
P1543	Dssrya_qF	ATCGGACAAGATGGCTCTGAC
P1544	Dssrya_qR	GCATGGCCTCGTTTAAGAAGGA
P1547	Dsnullo_qF	GCTGAAAATGTGAAGAGCGGAG
P1548	Dsnullo_qR	CTTTTGCTTCCTGGCCGAAATG
P1551	Dsbnk_qF	CTGACCAACACCTTTGAGTCG
P1552	Dsbnk_qR	AAAAGACTGTTTGCCACGCC
