# Supplementary material for: Identification and characterization of four Drosophila suzukii cellularization genes and their promoters
Source: BMC Genet. 2020 Dec 18;21(Suppl 2):146. doi: 10.1186/s12863-020-00939-y (PMC7747377; doi:10.1186/s12863-020-00939-y)
Supplement: Supplementary file 2 — Additional file 2: Figure S1. Drosophila suzukii NULLO protein alignment. DsNULLO is aligned with orthologs from Drosophila biarmipes [DbNULLO] (GenBank: XP_016948821.1), Drosophila hydei [DhNULLO] (GenBank: XP_023161247.1), Drosophila kikkawai [DkNULLO] (GenBank: XP_017022675.1), Drosophila melanogaster [DmNULLO] (GenBank: NP_511067.3), Drosophila miranda [DmirNULLO] (GenBank: XP_017156218.1), Drosophila navojoa [DnNULLO] (GenBank: XP_017964581.1), Drosophila obscura [DoNULLO] (GenBank: XP_022210380.1), Drosophila rhopaloa [DrNULLO] (GenBank: XP_016982709.1), Drosophila serrata [DserNULLO] (GenBank: XP_020805612.1) and Drosophila willistoni [DwNULLO] (GenBank: XP_002071148.1). Identical amino acids are shaded black and conservative changes are indicated in gray. All proteins contain a consensus site for N-terminal myristoylation (M) followed by a positively charged cluster (P). The remainder of the protein contains five conserved regions of amino acids (A–E) separated by short non-conserved regions. Figure S2. Drosophila suzukii SERENDIPITY-α (SRY-α) protein alignment. DsSRY-α is aligned with orthologs from Drosophila grimshawi [DgSRY-α] (GenBank: XP_001995347.1), Drosophila hydei [DhSRY-α] (GenBank: XP_023168708.1), Drosophila melanogaster [DmSRY-α] (GenBank: NP_524580.1) and Drosophila virilis [DvSRY-α] (GenBank: XP_002056142.1). The putative transmembrane domain is underlined and the region of similarity with proteins of the ERM family is boxed. Figure S3. Drosophila suzukii BOTTLENECK (BNK) protein alignment. DsBNK is aligned with orthologs from Drosophila grimshawi [DgBNK] (GenBank: EDV90810.1), Drosophila hydei [DhBNK] (GenBank: XP_023168586), Drosophila kikkawai [DkBNK] (GenBank: XP_017034088.1), Drosophila melanogaster [DmBNK] (GenBank: NP_524604.2), Drosophila miranda [DmirBNK] (GenBank: XP_017140437.1), Drosophila obscura [DoBNK] (GenBank: XP_022213695.1), Drosophila virilis [DvirBNK] (GenBank: XP_002055918.1) and Drosophila willistoni [DwBNK] (GenBank: X [file 12863_2020_939_MOESM2_ESM.pdf]

Additional file 2. Supplementary figures S1-S9.

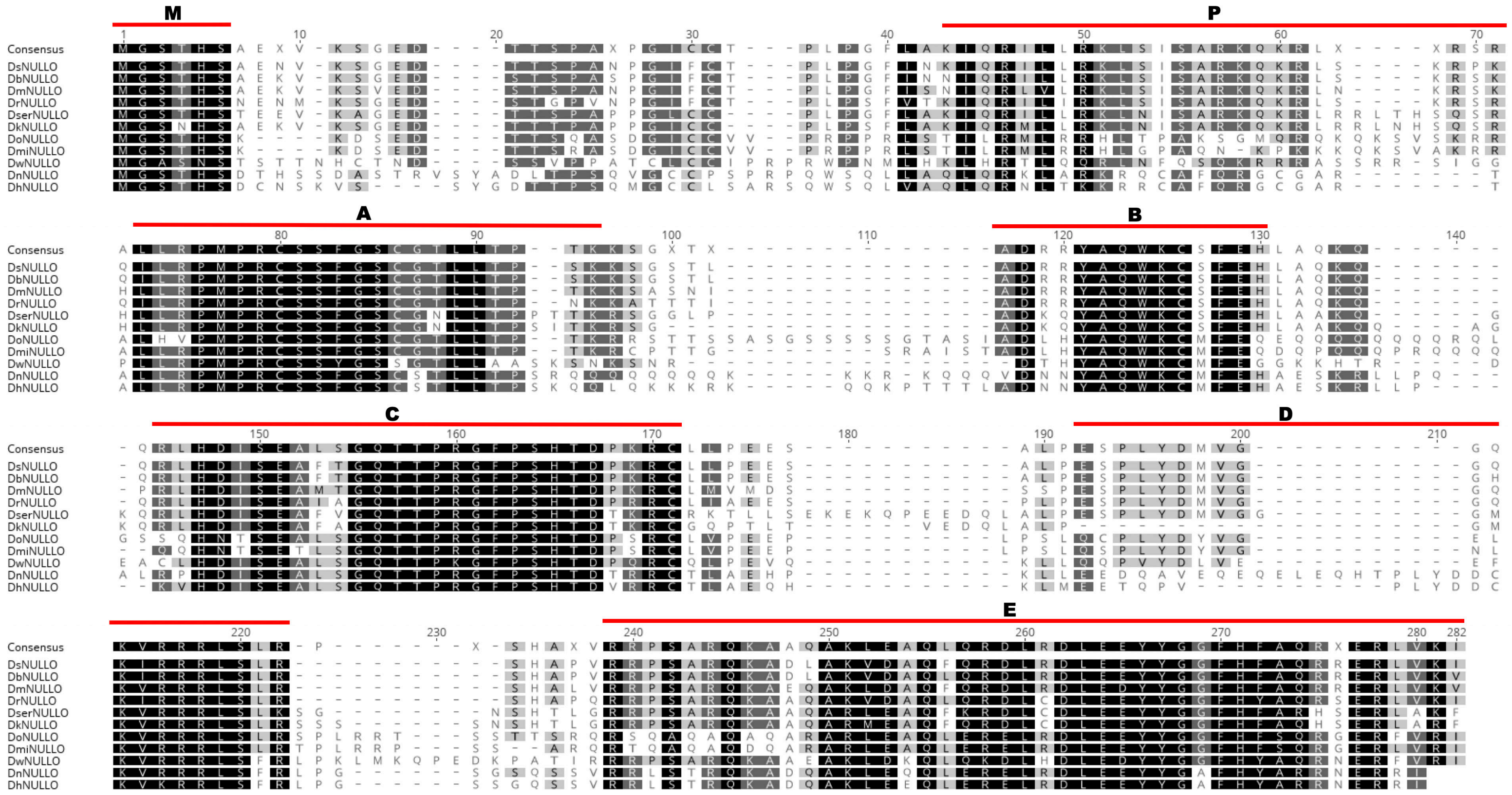

Figure S1. *Drosophila suzukii* NULLO protein alignment. DsNULLO is aligned with orthologs from *Drosophila biarmipes* [DbNULLO] (GenBank: XP_016948821.1), *Drosophila hydei* [DhNULLO] (GenBank: XP_023161247.1), *Drosophila kikkawai* [DkNULLO] (GenBank: XP_017022675.1), *Drosophila melanogaster* [DmNULLO] (GenBank: NP_511067.3), *Drosophila miranda* [DmirNULLO] (GenBank: XP_017156218.1), *Drosophila navojoa* [DnNULLO] (GenBank: XP_017964581.1), *Drosophila ob*scura [DoNULLO] (GenBank: XP_022210380.1), *Drosophila rhopaloa* [DrNULLO] (GenBank: XP_016982709.1), *Drosophila serrata* [DserNULLO] (GenBank: XP_020805612.1) and *Drosophila willistoni* [DwNULLO] (GenBank: XP_002071148.1). Identical amino acids are shaded black and conservative changes are indicated in gray. All proteins contain a consensus site for N-terminal myristoylation (M) followed by a positively charged cluster (P). The remainder of the protein contains five conserved regions of amino acids (A–E) separated by short non-conserved regions.

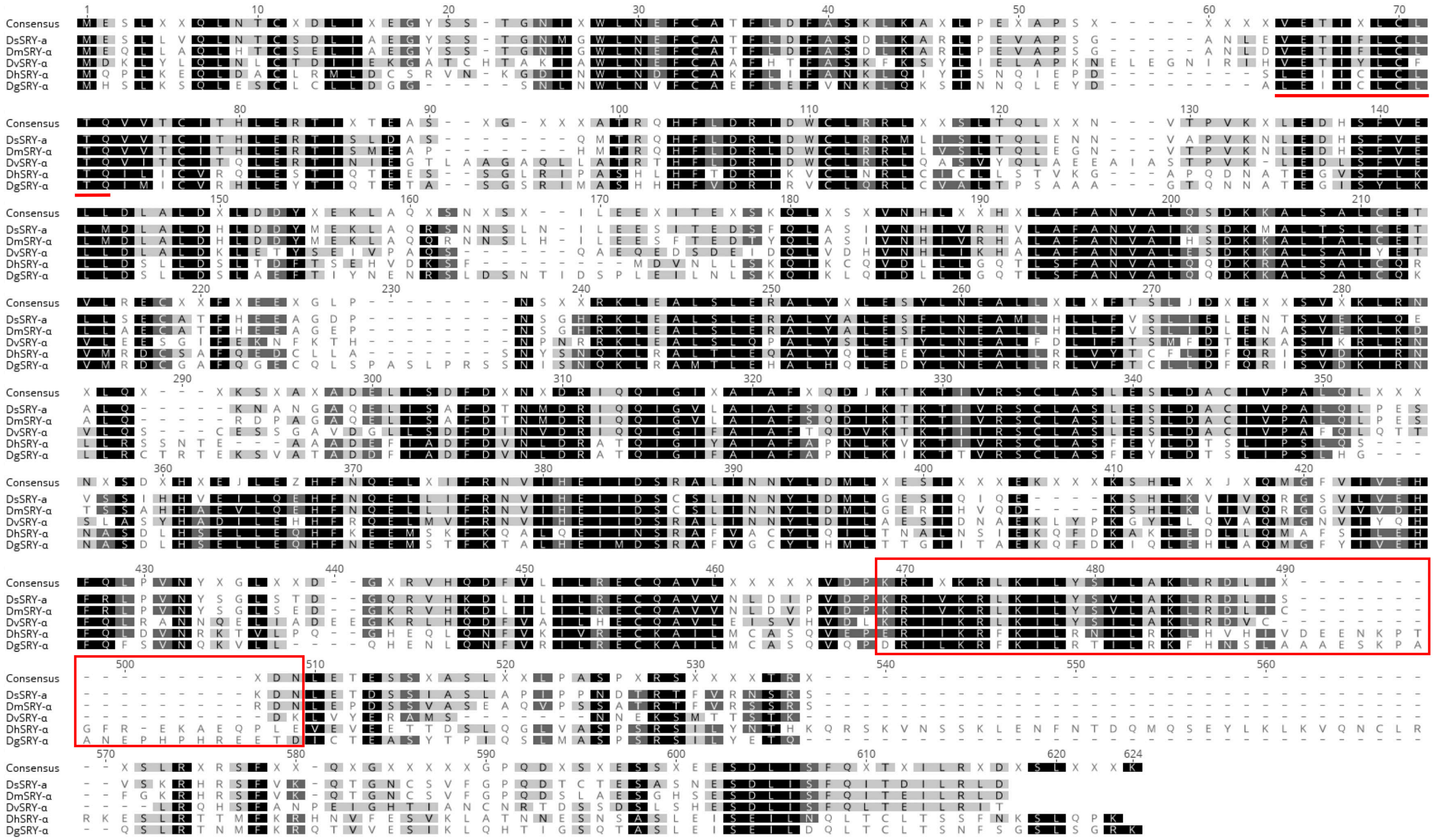

Figure S2. *Drosophila suzukii* SERENDIPITY-α (SRY-α) protein alignment. DsSRY-α is aligned with orthologs from *Drosophila grimshawi* [DgSRY-α] (GenBank: XP_001995347.1), *Drosophila hydei* [DhSRY-α] (GenBank: XP_023168708.1), *Drosophila melanogaster* [DmSRY-α] (GenBank: NP_524580.1) and *Drosophila virilis* [DvSRY-α] (GenBank: XP_002056142.1). The putative transmembrane domain is underlined and the region of similarity with proteins of the ERM family is boxed.

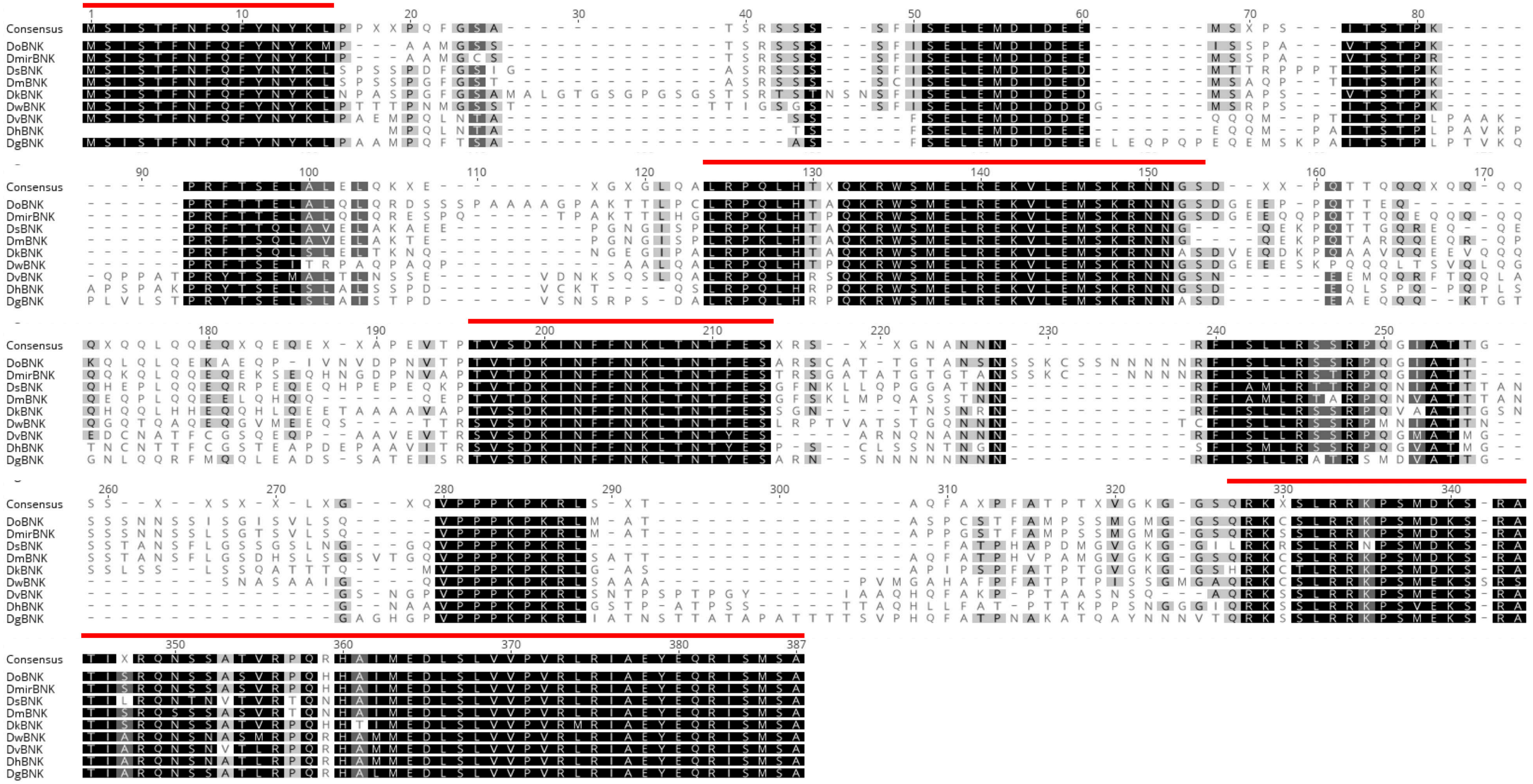

Figure S3. ***Drosophila suzukii* BOTTLENECK (BNK) protein alignment**. DsBNK is aligned with orthologs from *Drosophila grimshawi* [DgBNK] (GenBank: EDV90810.1), *Drosophila hydei* [DhBNK] (GenBank: XP_023168586), *Drosophila kikkawai* [DkBNK] (GenBank: XP_017034088.1), *Drosophila melanogaster* [DmBNK] (GenBank: NP_524604.2), *Drosophila miranda* [DmirBNK] (GenBank: XP_017140437.1), *Drosophila obscura* [DoBNK] (GenBank: XP_022213695.1), *Drosophila virilis* [DvirBNK] (GenBank: XP_002055918.1) and *Drosophila willistoni* [DwBNK] (GenBank: XP_002072000.1). Highly conserved regions are marked with red lines.

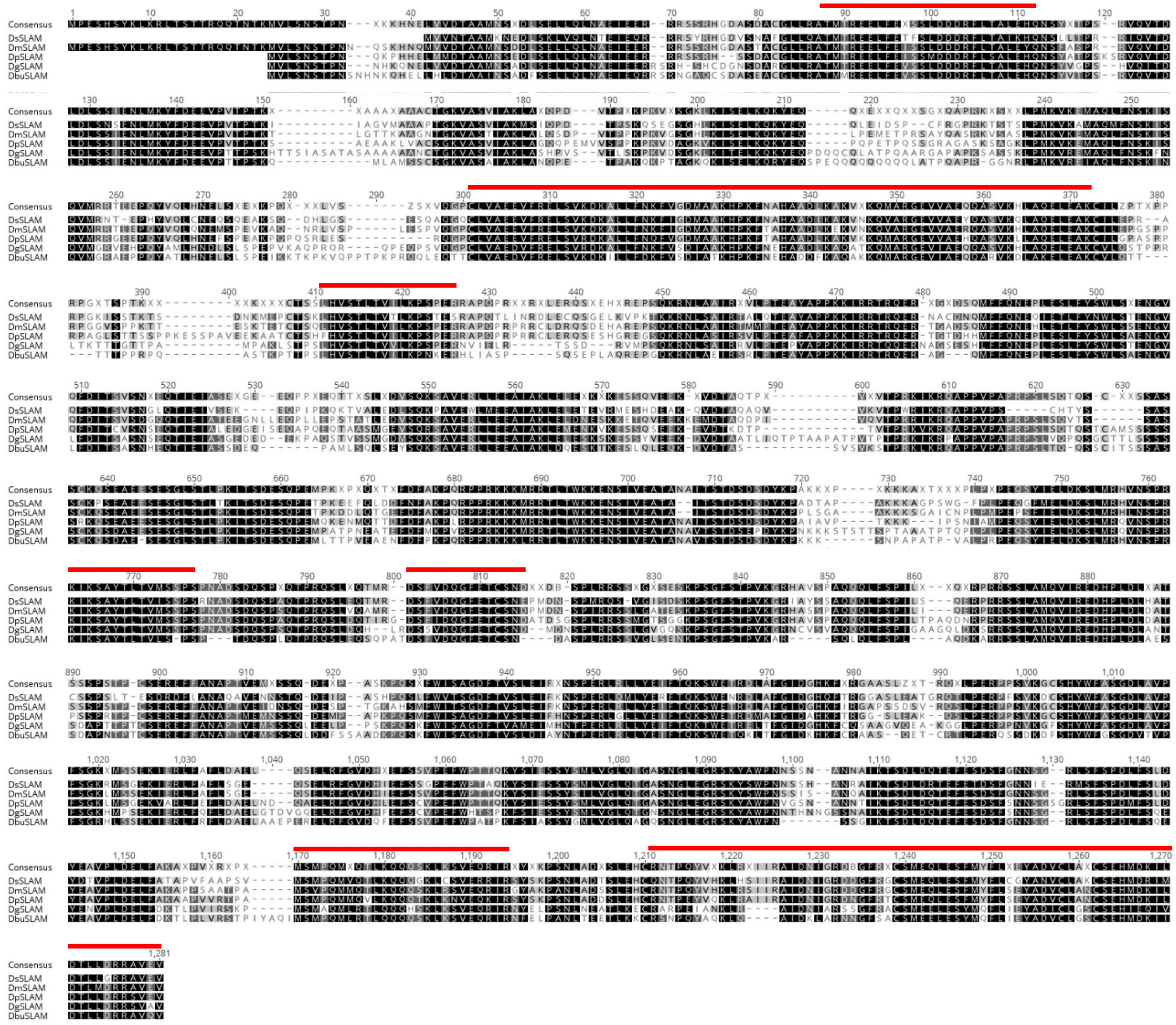

Figure S4. ***Drosophila suzukii*** **SLOW-AS-MOLASSES (SLAM) protein alignment**. DsSLAM is aligned with orthologs from *Drosophila grimshawi* [DgSLAM] (GenBank: XP_001988112.1), *Drosophila pseudoobscura* [DpSLAM] (GenBank: XP_001355861.2), *Drosophila melanogaster* [DmSLAM] (GenBank: NP_001285668.1), and *Drosophila busckii* [DbuSLAM] (GenBank: ALC38995.1). Highly conserved regions are marked with red lines.

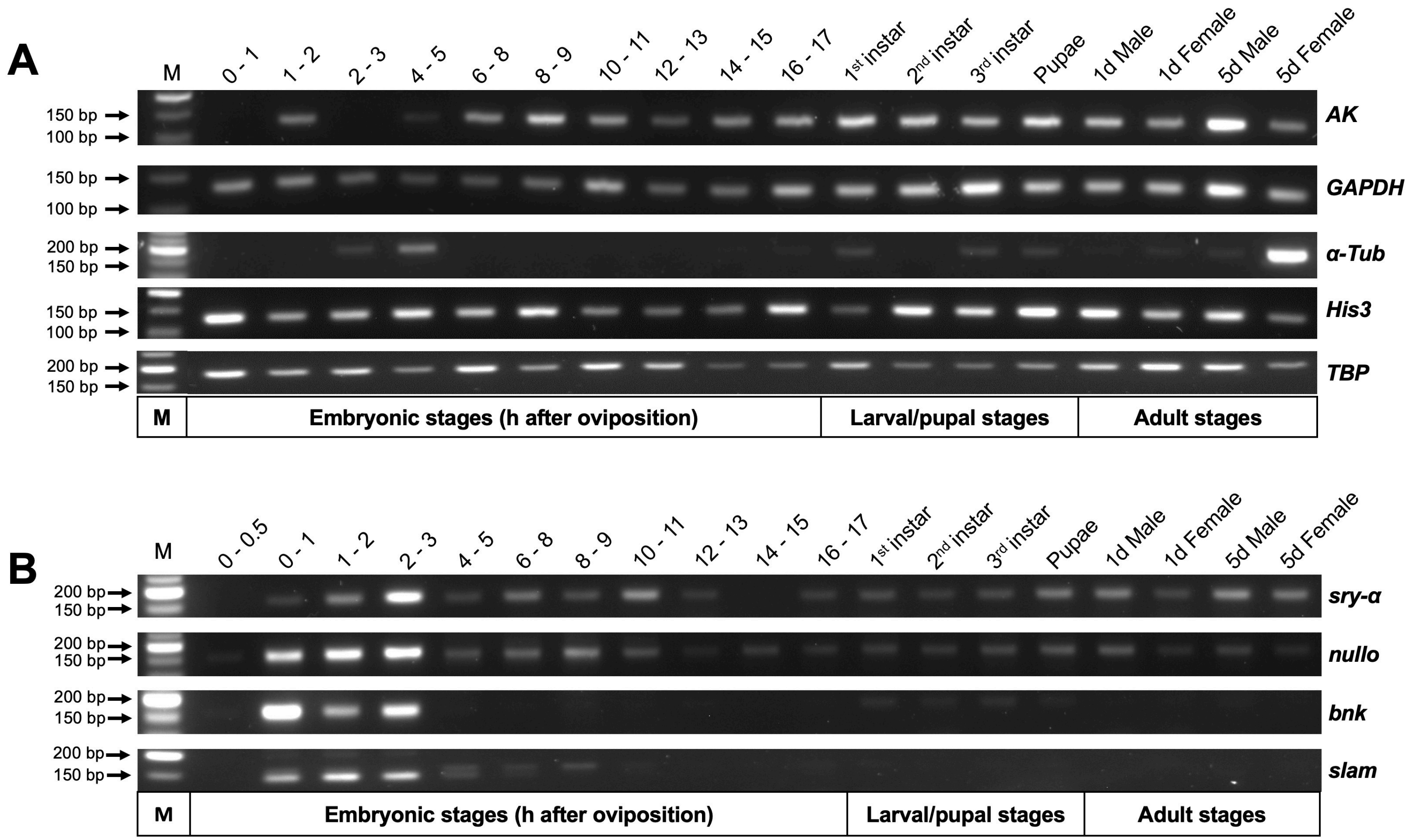

Figure S5. Reverse-transcriptase (RT)-PCR to evaluate the reference (a) and cellularization (b) genes through the development of *Drosophila suzukii*. Primer sequences for reference genes *TATA binding protein* (*TBP*), *glyceraldehyde-3-phosphate dehydrogenase* (*GAPDH*), *arginine kinase* (*AK*), *a-Tubulin* (*α-Tub*) and *Histone H3* (*His3*) (Zhai et al., 2014; Li and Handler, 2017), as well as for four cellularization genes can be found in "Additional file 1". Embryos collected at different time points after egg laying (in hours), larvae (first, second and third instar), pupae (2 days after prepupae), adult female and male (1 and 5 days old). Additional 0-0.5 h samples were used for cellularization genes. The PCR product sizes are 140 bp for *AK*, 130 bp for *GAPDH*, 189 bp for *α-Tub*, 129 bp for *His3*, and 182 bp for *TBP*, 161 bp for *sry-a*, 155 bp for *nullo*, 159 bp for *bnk*, and 149 bp for *slam*. M is the molecular weight ladder.

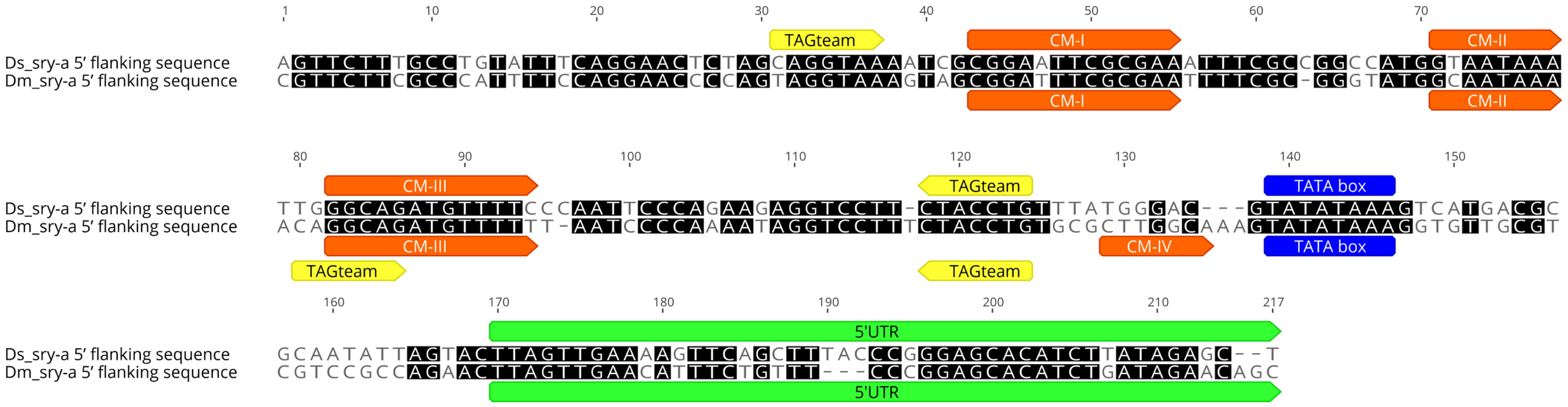

Figure S6. Alignment of the *Ds_sry-α* and *Dm_sry-α* 5' flanking sequences. The upstream flanking sequence from *Ds_sry-α* (165 bp, before it reaches the upstream gene DS10_00012896, the ortholog of *D. melanogaster janus A*) and *Dm_sry-α* (167 bp) together with the 5'-UTR (annotated in green) are compared. The 5' flanking sequences of *Ds_sry-α* and *Dm_sry-α* contain three and four conserved motifs that confer blastoderm-specific expression, respectively. Both 5' flanking sequences contain two TAGteam motifs (annotated in yellow), and a TATA box (TATATAAA) 23 bp upstream of the putative transcription start site. The *Ds_sry-α* 5' flanking sequence was fused to DsRed-NLS in V205 to act as a gene promoter for the *in vitro* test.

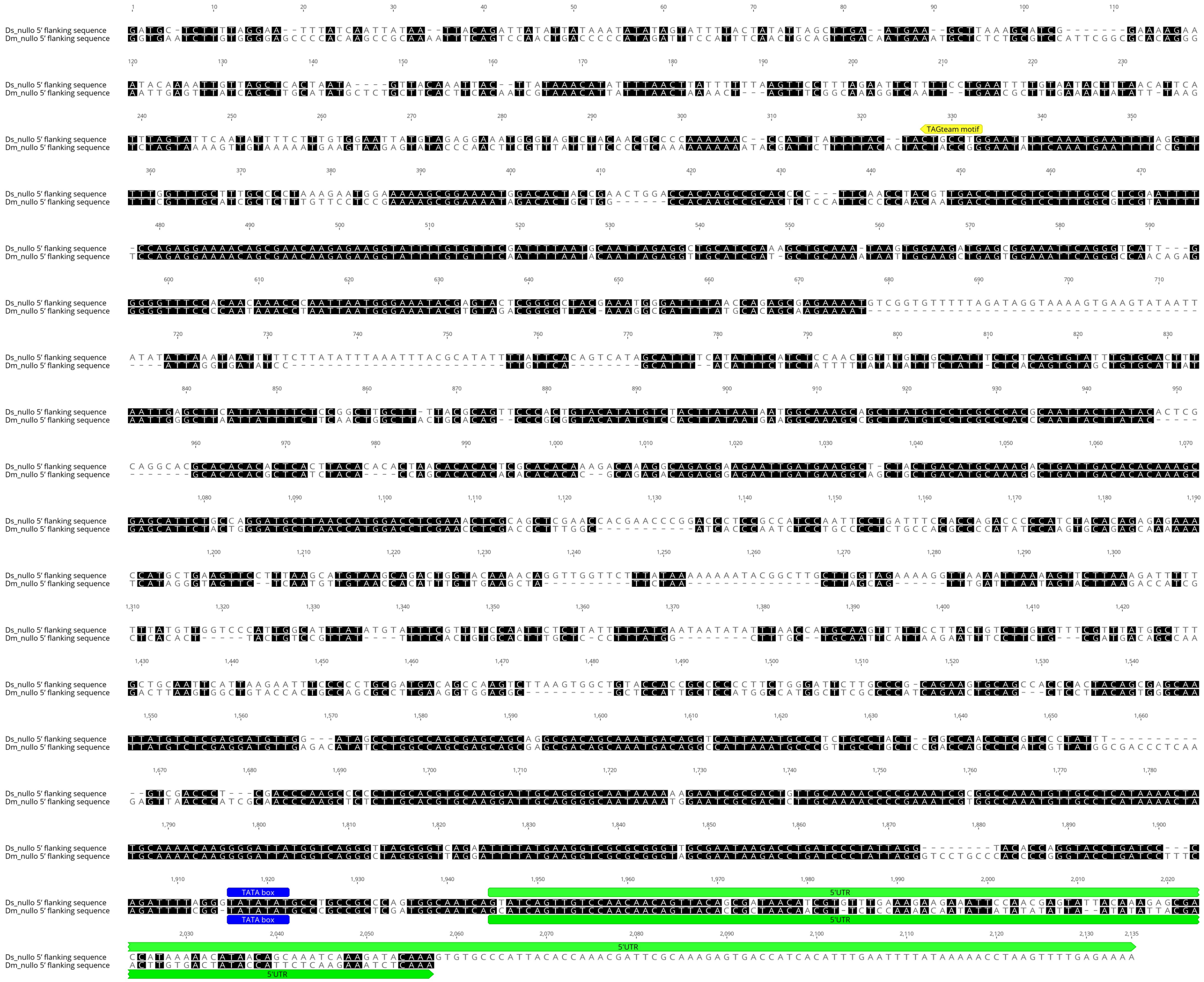

Figure S7. Alignment of the *Ds_nullo* and *Dm_nullo* 5' flanking sequences. The upstream flanking sequence from *Ds_nullo* (1876 bp) and *Dm_nullo* (1753 bp) together with the 5'-UTR (annotated in green) are compared. The 5' flanking sequences of *Ds_nullo* sequence contains one TAGteam motif (annotated in yellow), whereas that of *Dm_nullo* contains none. The TATA box (TATATAT) is 24 bp upstream of the putative transcription start site. The *Ds_nullo* 5' flanking sequence was fused to DsRed-NLS in V206 to act as a gene promoter for the *in vitro* test.

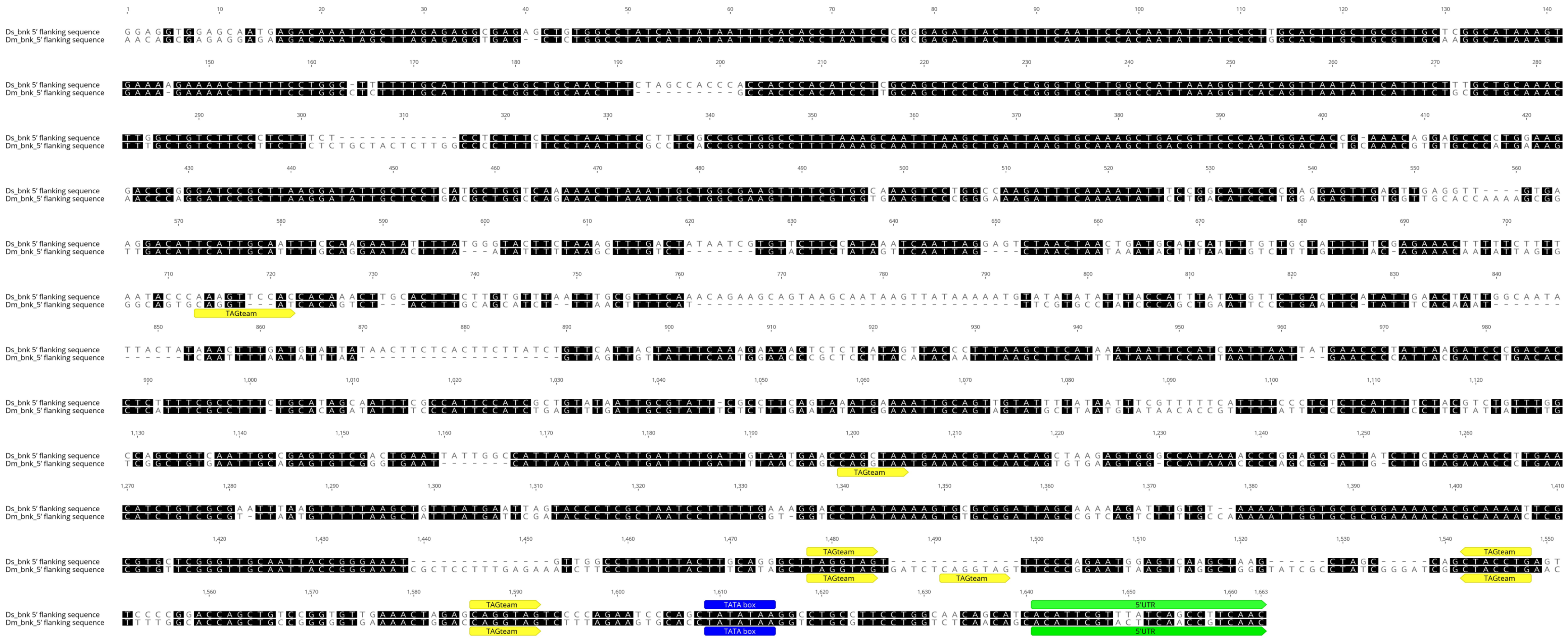

Figure S8. Alignment of the *Ds_bnk* and *Dm_bnk* 5' flanking sequences. The upstream flanking sequence from *Ds_bnk* (1581 bp) and *Dm_bnk* (1523 bp) together with the 5' UTR (annotated in green) are compared. The 5' flanking sequences of *Ds_bnk* and *Dm_bnk* contain three and four TAGteam motifs (annotated in yellow), respectively, and the TATA box (TATATAAA) is 25 bp upstream of the putative transcription start site. The *Ds_bnk* 5' flanking sequence was fused to DsRed-NLS in V208 to act as a gene promoter for the *in vitro* test.

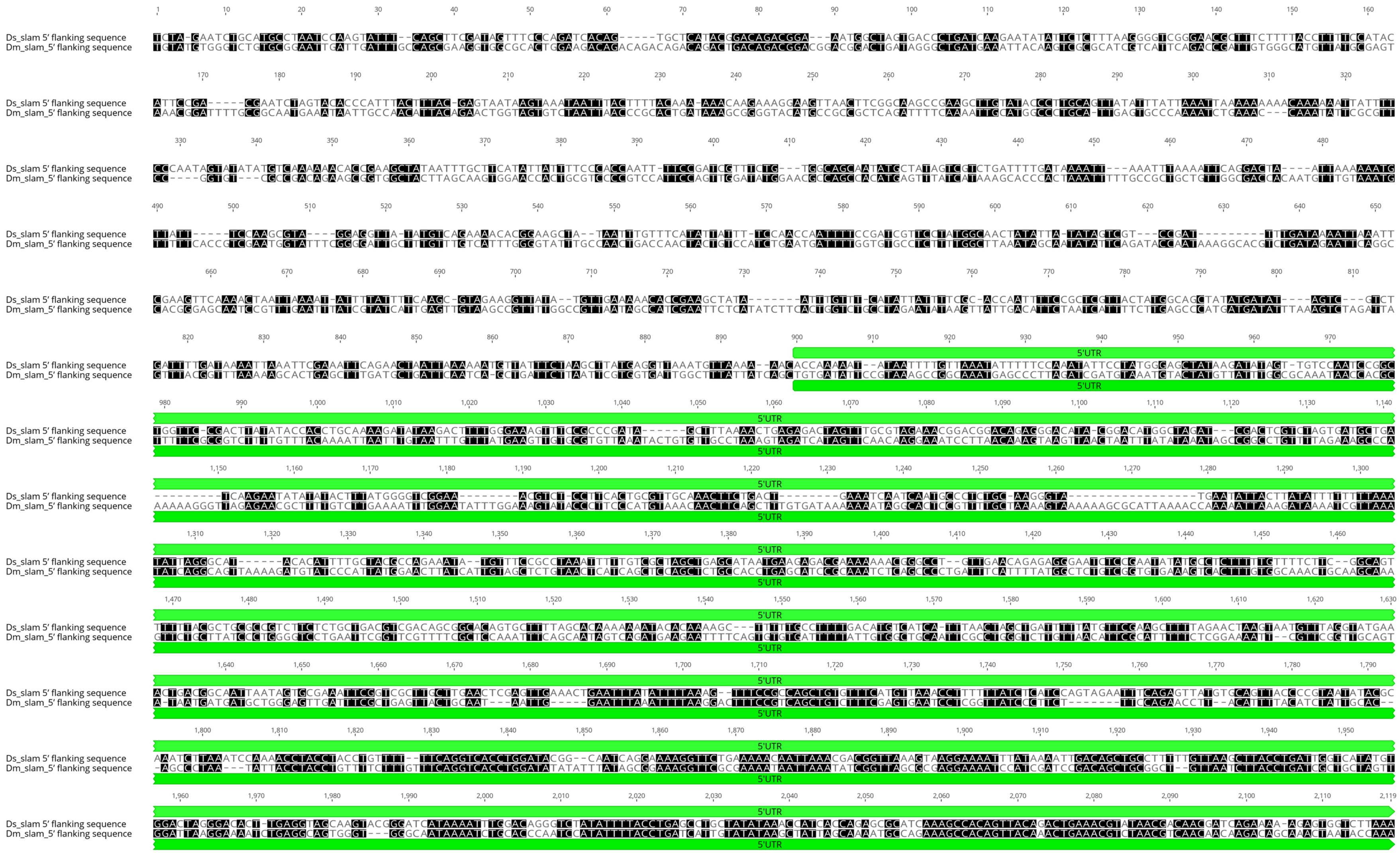

Figure S9. Alignment of the *Ds_slam* and *Dm_slam* 5' flanking sequences. The upstream flanking sequence from *Ds_slam* (822 bp) and *Dm_slam* (887 bp) together with the 5' UTRs (annotated in green) are compared. Neither the *Ds_slam* nor *Dm_slam* 5' flanking sequences contain TAGteam motifs or a TATA box. The *Ds_slam* 5' flanking sequence was fused to DsRed-NLS in V207 to act as a gene promoter for the *in vitro* test.
